# Emotional and Cognitive Responses to Theatrical Representations of Aggressive Behavior

**DOI:** 10.3389/fpsyg.2020.01785

**Published:** 2020-08-14

**Authors:** Alexandru I. Berceanu, Silviu Matu, Bianca I. Macavei

**Affiliations:** ^1^Laboratory for Cognitive Development and Applied Psychology through Immersive Experiences, CINETic Centre, National University of Theatre and Film “Ion Luca Caragiale”, Bucharest, Romania; ^2^Department of Clinical Psychology and Psychotherapy, Babeş-Bolyai University, Cluj-Napoca, Romania; ^3^Department of Psychology, Babeş-Bolyai University, Cluj-Napoca, Romania

**Keywords:** theater, violence, aggressive behavior, emotion regulation, embodiment, theater therapy

## Abstract

Representation of human conflict is central to theater performance. In our study, we have used self-reported measures of emotional experience and a word recall task, in order to assess the effects of theatrical representations of violence. Forty participants were randomly assigned to the role of performer or spectator, in either a realistic representation of a script or in a version that recollected the same actions of the script in a series of intertwined monologs. The script represented an aggressive interaction between two work colleagues. Our results show both statistically significant differences in the levels of depression and positive emotions reported after enacting the script, as well as differences in the performance on the word recall task containing aggressive related and non-related stimuli. The results point to stronger effects for performing theatrical representations of violent actions, as compared to recollecting or watching such actions. The fact that actors experienced higher positive emotions is in line with the two-pedal model of aggressive behavior. This model suggests that negative emotions toward aggressive behavior might change to positive emotions due to the repetition in performing violent behaviors, as a key for the transition from reactive aggression to appetitive aggression. Other implications for the study of aggression in theatrical representations are discussed.

## Introduction

Representation of human aggressive behavior is central to theater since its inception. Almost always, dramatic action is based on the representations of conflict. In dramatic theater, the confrontation of two entities is almost always violent, either at the physical level or, very often, at the psychological level. The antagonist acts as an opposing force and prevents the protagonist from attaining his goal (Aristotel, 1957). Aggressive behavior refers to behavior meant to injure or irritate another person (Berkowitz, 1993). One of the main models for human aggressive behavior is the frustration–aggression model, where frustration arises from an obstacle to reach a goal and elicits an aggressive behavior (Dollard et al., 1939; Berkowitz, 1990, DiGiuseppe and Tafrate, 2007). From the perspective of the frustration–aggression model, we can see the development of the theatrical action into violent behavior as arising from the frustration of the protagonist and the antagonist. The social function of violence representation was stated in antiquity by Aristotle through catharsis and in modern times by René Girard through his concept of the scapegoat (Girard, 1995). Taking into account Bandura’s theory on vicarious learning (Bandura, 1983, 2001), strong questions arise around the social benefits of theatrical representations of violence.

Nowadays, violence is an attraction just as much as it was in the past. For many performative productions, from commercial ones to performances addressed to a highly specialized public, exposure to violence is a constant. From an evolutionary perspective, the ordeal hypothesis regards the representation of violent acts in fiction as a simulation ability developed through natural selection, which enables humans to learn behaviors required in dangerous situations, without the risk of facing their consequences through direct exposure (Morin et al., 2019). While readers experience violent actions only through vicarious imagination, spectators watch violent actions and actors perform them.

### Theatrical Representation as Experimental Tool in Exploring Violent Behavior

Theater offers ecological conditions for studying aggressive behavior while at the same time providing controlled and ethical conditions, as well as spontaneity and realism. Moreover, role playing has been previously used as an efficient instrument for inducing aggressive-like behaviors (Moran et al., 2014). Theater play offers an immersive perspective for actors, which makes it suitable as a highly experiential tool for interventions. Some schools of psychotherapy (e.g., psychodrama) have a long history of using theater-inspired experiences for treating psychological problems (Baim, 2007; Baim et al., 2007).

One of the main reasons for which theater has not yet become a common focal point for scientific research is the innate complexity of theatrical actions, which causes difficulties in manipulating specific variables in order to isolate components, such as role play, imaginary action, physical action, etc. There are a lot of different approaches in theatrical representation that bring multiple stances of self, action, and reality. In Grotowski’s perspective on the body as an instrument (Grotowski, 2002), there is a complete overlap between the actor’s biological being and the fictional body. In the case of puppet performance, there is a strong separation between the actor and the body that performs the action. Studies show that agent perspective on the performed action triggered by role play is accompanied by differences at the functional level of the brain (Brown et al., 2019) and has effects on opinion change (Janis and King, 1954; King and Janis, 1956).

Actors might enact a scene of violence multiple times. Playing a successful Othello, one could perform the strangulation of Desdemona more than 100 times. While the ordeal hypothesis indicates a social function of experiencing violence through fiction, the emotional consequences for the performers of such scenes are largely unknown. Lucy Nevitt states that simulated violence, while producing no physical harm, is so connected to reality that it cannot be easily assigned as “not real” (Nevitt, 2013).

### Theatrical Representation Type and Level of Immersion

In this study, we focus on how violence affects spectators and performers of violent acts in two types of representations: performed violence (PV) and suggested violence (SV). This is a long-standing dividing line in the esthetics of theatrical representation in the European theater tradition, with two extreme positions. In the Greek tragedy, the actual violent acts were not represented on stage, while in Roman spectacles, you could have actual killings happening on stage (Heinrichs, 2000; Sommerstein, 2010; Peachin, 2011). In our study, the actors performed the violent actions of the script in the PV condition in a realistic representation. In the SV condition, they performed intertwined monologs recollecting the same actions in a dyadic interaction. Each representation was watched by two student actors.

We can assume that the two types of representations have a different impact on actors, as the two types of immersion in fiction that they promote are very different: in SV, actors rely mainly on imagination and autobiographical memory, while in PV, they rely on imagination, autobiographical memory, as well as sensations and emotions driven by the real stimuli from rehearsals. Since there are strong differences in the processes involved in the two types of theatrical performances, we think that the emotional experience of an actor performing an action should be of a different kind from the one recollecting the action. The experience for the actor actually performing the action might be more immersive, resulting in a stronger emotional response. Similarly, spectators of PV would have a more immersive experience observing aggressive acts than spectators of SV. In our study, we want to investigate if the two different types of theatrical representations of aggressive actions, PV and SV have different effects on performers and spectators at the emotional and cognitive levels.

## Materials and Methods

In order to assess the effects of theatrical representations of violent actions on performers and spectators based on the type of representation, SV vs. PV, we developed a script based on a violent interaction in a work conflict between two colleagues. The script was especially designed for the experiment, and it followed the same units of action and timing both in SV and PV.

### Script

Dan and Victor are colleagues sharing the same office. Dan forces Victor to stay at work after hours, in order to finish an important project for the company. Dan bullies Victor verbally, psychologically, and physically, finally locking him in the room. While alone, Victor destroys the project they were working on by deleting the file from the computer. Dan is outraged and destroys Victor’s brand new laptop. Victor attacks Dan and stabs him with a pencil and then throws hot coffee on him. The script provided obstacles for each of the characters in attaining a goal that seemed reachable, gradually building the escalation of conflict to aggressive behavior, in accordance with the frustration–aggression model. The script was adjusted to performers gender performers by changing the names accordingly.

The script had a wide range of violent actions: verbal humiliation, verbal aggression, aggressive behavior oriented toward objects, and physical assault. Due to the gradual escalation of conflict, the overall perspective of the script is ecological, providing the participants with a realistic setup.

### Objectives

Our study aimed to investigate how the two different types of theatrical representation of violence, PV and SV affect performers and spectators at the emotional and cognitive levels, based on the different levels of immersion they provide. Understanding the effects of theatrical representations of violence will contribute to further developing theater-based interventions for regulating aggressive behavior and to establishing an ecological and ethical approach to studying human aggressive behavior.

The very first goal of this study was to investigate how assuming different agency stances in an aggression-related event could impact emotion and memory. Subjects directly performed an aggressive behavior (performer, PV), talked about an aggressive behavior (performer, SV), observed an aggressive behavior (spectator, PV), or heard somebody talking about an aggressive behavior (spectator, SV). The notion of embodiment refers to the assumption that thoughts, feelings, and behaviors are grounded in bodily states and sensory experiences (Niedenthal et al., 2005; Barsalou, 2008). In accordance with the concept of *embodiment*, we expected that a different level of physical involvement in the representation of aggressive action would be accompanied by differentiated effects at emotional and cognitive levels.

Our hypothesis is that, overall, the representation of aggressive actions will have a stronger impact on the subjects in PV condition than on those in SV, as well as on the subjects in performer condition compared to the ones in the spectator condition, at emotional and cognitive levels.

From an information processing point of view, the role of cognition in aggressive behavior can be explored considering the notion of cognitive schemas: self-schemas, normative beliefs, world schemas, and scripts (Huesmann, 1998). The more extensive and primed the networks are, the more accessible they become (Huesmann, 1998). In addition, self-schemas (i.e., the terms in which one defines oneself) and world schemas (i.e., the terms in which one defines the world around) can facilitate or inhibit further activation of aggression-related scripts. The scripts and schemas one has developed are shaped and reinforced into one’s long-term memory through observational and enactive learning (Huesmann, 1998). Semantic priming can be used to investigate the impact or accessibility of a word preceded by another semantically related word or situation (Neely, 1991; McNamara, 2005).

In our study, we investigated the impact of activating violence-related cognitive schemas on word recall in an explicit memory task. Word recall tasks can be used to investigate the ways in which the activation and selection of cognitive schemas and scripts impact information retention and retrieval (Eysenck and Byrne, 1994; White et al., 2014). The activation of an aggression-related schema can facilitate the retention and retrieval of aggression-related verbal material. In addition, an active aggression-related schema may lead to the false recall of aggression-related, previously non-studied words. Thus, the activation of violence-related schemas was operationalized as the efficient learning and processing of violence-related material and the precarious learning of violence irrelevant material. Results in word recall list tasks were previously associated with trait aggressive behavior scores, as well as with different types of priming over cognitive schemas, through various types of exposure to aggressive behavior (Vannucci et al., 2012).

### Participants

Forty undergraduate students of different expertise levels (first, second, and third year of study) participated in our study (30 female, 10 male). All were acting students from two different Romanian theater universities. All of them were native Romanian speakers. The two groups from the two universities were not mixed during the experiment. Participation in the study was voluntary, with no financial or educational incentives. The procedure was approved by responsible ethics committees.

After signing informed consent forms, participants were assigned through block randomization to a role in the experiment (performer or spectator), a type of representation (either PV or SV), and a part in the script (Victor or Dan/Victoria or Dana). Separate randomizations procedures were performed in the two universities where the experiments took place. The lines of the script were adapted to the gender of the actor. This procedure resulted in 10 actors in the PV condition, 10 actors in the SV condition, and 20 spectators, 10 in each type of representation.

### Design

After block randomization, students in “actor” roles had 2 days of rehearsals. On the third day of the experiment, subjects assigned as “actors” performed the rehearsed scene, and the subjects assigned as “spectators” watched the performance (either SV or PV). Just before the performance, both actors and spectators filled a pretest questionnaire asking about their current mood. Just after the end of the performance, subjects filled posttest questionnaires, including the same scale asking about current mood and items investigating emotional reactions to the performed script. Finally, they performed the word recall task (see section “Instruments”).

### Rehearsals and Presentation

All actors had the same amount of time for rehearsals: day 1–20 min reading, blocking for 1 h; day 2–rehearsal of two run-throughs of ∼20 min each; day 3–subjects in the performer condition performed rehearsed pieces (SV or PV) for subjects in the spectator condition; each pair of actors was watched while performing by a pair of spectators. Blocking rehearsal provided main movements requested by the script to be done by the actors.

Actors in the PV condition were presented with the blocking and then rehearsed it. Suggestions in changing the blocking were accepted if they were suitable for the progression of the action. Blocking was limited to the minimum necessary for the action to advance (e.g., getting up, locking door, hitting laptop). Actors in SV had the same props as actors in PV on their desk. Actors in the SV condition were seated at a long table facing each other and were instructed to relate through eye contact during monlogues. We believe that this type of interaction serves at comparing the two conditions, PV and SV, as actors in both were part of a dyadic interaction.

The same daily schedule was used for SV and PV. All actors had the same directing notes, with actors in PV also having blocking directions. All participants were asked not to disclose the subject of the play to their colleagues. The actors only participated in their own rehearsals and were asked not to give feedback to their partners. There was no qualitative feedback given to the actors, and no evaluation of the characters in the play was made. The approximate duration for all performances was ∼10 min, in both SV and PV.

During the presentation, some of the actors in the SV condition told parts of the monologs in the audience’s direction. None of the presentations were stopped, and they all succeeded to go through. Most of the performances followed the script; however, some of them missed some lines or actions in the performance, but were not asked to redo the action. Differences from the script were not considered significant with regard to the duration and importance of the performed actions by an external and an internal evaluator.

### Instruments

#### The Profile of Affective Distress PDA

The Profile of Affective Distress (Opris and Macavei, 2007a) is a 39-item questionnaire consisting of words that describe positive and negative emotions. The questionnaire was based on the shortened version of the Profile of Mood States (POMS, DiLorenzo et al., 1999). The construction of the questionnaire was based on a previous study (Opris and Macavei, 2005) that investigated the qualitative differences between functional and dysfunctional negative emotions, providing evidence in support of the binary model of distress (Ellis, 1994). The instrument allows the calculation of separate scores for negative and positive emotions, negative functional and negative dysfunctional emotions, as well as distinct emotions, namely, sadness, depression, worry and anxiety, anger, and annoyance (Opris and Macavei, 2007a, b). Four additional items were added, in order to comprise the entire hostility scale from the Positive and Negative Affective Schedule (PANAS; Watson and Clark, 1999), which was also used as an individual scale in our study. All items are scored on a 5-point Likert scale, with higher scores indicating higher levels of experiencing a particular emotion. The questionnaire is reported to have good psychometric properties. Internal consistency is high, with a Cronbach’s alpha of 0.94 (Opris and Macavei, 2007b). Content and construct validity were also assessed, proving that the questionnaire is a valid measure of affective distress, as well as negative and positive emotions (Opris and Macavei, 2007a, b). The hostility scale of the PANAS has also shown very good psychometric properties, with a Cronbach’s alpha of ∼0.80 or above (Watson and Clark, 1999).

#### Presence Questionnaire

The presence questionnaire (PQ) (Witmer and Singer, 1994) was originally developed to assess the experience of presence in virtual environments, defined as the subjective experience of being in one place or environment, even if one is physically situated in another (Witmer and Singer, 1994, 1998). The presence questionnaire has 28 items on eight dimensions. We adapted the questionnaire to the context of a theater performance. Participants indicated on a 0–7 Likert type scale from “not at all” to “very much,” the degree to which they felt the experience of being in the place and context described by the play. The questionnaire items check for realism of action, visual aspects, sounds, involvement, distraction, time awareness, and distancing/closeness from action and characters.

#### Word Recall Task

To assess the activation of violence-related schemas, we created a word recall task. The task was administered at ∼5 min after the end of the representation, at the same time for both spectators and performers. The task consisted of intentionally memorizing a list of 45 words from three lexical families: violence/aggression (15 words), peacefulness (15 words), and neutral words that did not fall into any of the previous categories (15 words). Each subject was asked to read the list of words at a normal pace (the words from the three lexical families were presented in a mixed order) and to try to remember as many as possible. Immediately afterward, the participants were required to perform a set of simple mathematical calculations for 2 min (e.g., 20 + 32=; 24 − 32=). The distraction procedure was meant to interfere with working memory, so that the effects of active cognitive schemas could be observed. After the distraction task, the subjects were asked to write down all the words they could recall from the initial list, in no particular order. This procedure allowed us to explore the effects of the role and the type of play on the activation of cognitive schemas related to violence. We subsequently counted the number of words recalled from each category, as well as words that were not previously presented (i.e., falling into a false memory category).

### Assessing the Actions in the Script That Elicited the Most Intense Emotions

Three items were created to estimate which actions from the script elicited the most intense emotions. The first item asked the subject to name the action or the moment in the script/play that had elicited the most intense negative emotion. Subsequently, a list of 13 negative emotions was provided (fear, anger, guilt, shame, embarrassment, hurt, sadness, annoyance, depression, worry, envy, jealousy, contempt) on which the respondent could check the identified emotion and rate its intensity on a 0–10 Likert type scale. A 14th position was left empty for the subject to fill in, in case he/she felt something else. A second item was created to give the subjects the possibility to name the most important moments during the play when they experienced the most intense negative or positive emotions. The respondents had to describe the top 5 relevant moments, to name the emotion associated with each one, and to rate the intensity of that emotion on a 0–10 Likert scale. The third item described seven of the most relevant aggressive moments in the script and asked the subjects to rank them from the most to the least intense. The seven moments were (1) Dan repeatedly slaps Victor’s face, (2) throwing hot coffee on Dan’s face, (3) locking Victor in the room against his/her will, (4) destroying the laptop, (5) verbally humiliating Victor, (6) stabbing Dan’s arm with a pencil, and (7) grabbing and eating Victor’s food. For each of these moments, the participants had to name the main emotion they felt and rate its intensity on a 0–10 Likert type scale.

These three items were created to help estimate the moments during the play at which the participants felt the most intense emotions. In addition, the items allowed respondents to express both negative and positive emotions in relation to aggressive behaviors.

The choice of statistical methods we used in our analysis was guided not only by the type of data that we collected but also by the degree to which our data met the statistical assumptions required for a particular analysis. For continuous and normally distributed data, we preferred parametric analysis. However, if normality assumptions for our distributions were not met, we reported the results of non-parametric statistics. To reduce type I error due to multiple comparisons between groups and subgroups, and across multiple dependent variables, we used the procedure described by Benjamini and Hochberg (1995) to control the false discovery rate (FDR). For each family of hypotheses, we only considered as statistically significant the results of those tests with an associated *p-*value smaller than the largest *p* falling below the critical value calculated with the Benjamini and Hochberg (1995) procedure (B–H). This critical value was calculated as (*i*/*m*) × *Q*, where *i* is the rank of the *p*-value in an ascending ordered list of all *p*-values, *m* is the total number of comparisons, and *Q* is the value of the chosen FDR, which, in our case, was 0.05. All *p*-values smaller than the largest one that fell below the corresponding B–H critical value were considered significant, even if they were larger than their specific critical values. For non-parametric statistics, in order to analyze the interaction between type of play and role in the experiment (performed vs. simulated play X actor vs. spectator role), we first performed an omnibus test comparing all four subgroups of the interaction term. We performed pairwise comparisons of the subgroups only if the probability associated with the omnibus test was below the standard two-tailed α = 0.05. The *p*-value for the omnibus test was not taken into account in the B–H procedure, but the values for subsequent pairwise comparisons were considered together with the results for the main effects for type of play and role in the experiment.

## Results

### Impact of Play on Emotional Experience

We looked at the impact that the type of play (performed vs. simulated) and role (actor vs. spectator) had on the emotional experience of the participants, by analyzing mood changes on the standardized items of the PDA + and the additional one for assessing hostility from PANAS. Change scores for each scale (depression, sadness, fear, worry, anger, annoyance, hostility, and positive affect) were computed as posttest *minus* (-) pretest scores and compared using non-parametric tests. The B–H procedure, as described above, was applied to all pairwise comparisons, across all emotion scales.

**FIGURE 1:**
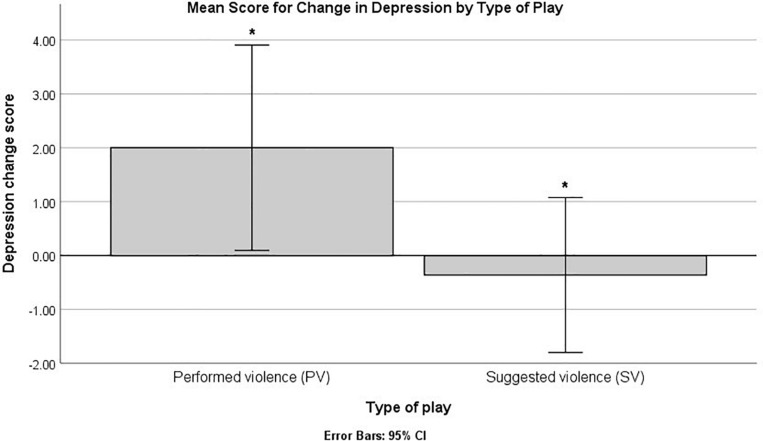
Mean scores for depression change scores by type of play. The bars marked by “*” indicate statistically significant differences based on a non-parametric analysis and B–H procedure for controlling false discovery rate (FDR).

**FIGURE 2:**
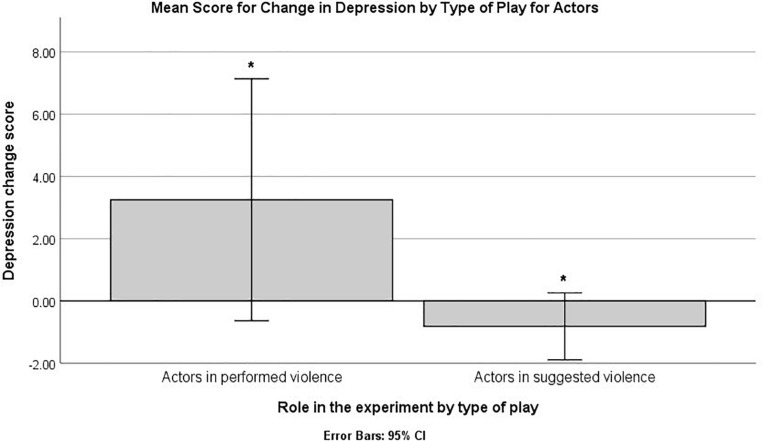
Mean scores for depression change scores by type of play for actors in the experiment. The bars marked by “*” indicate statistically significant differences based on a non-parametric analysis and B–H procedure for controlling false discovery rate (FDR).

Non-parametric between-groups comparisons using the Mann–Whitney test on the change scores identified a significant difference on depression, when comparing the type of play (main effect of performed vs. suggested violence; see Figure 1). Higher ranks (indicating higher scores) were identified for the performed play, *U* = 87.00, *Z* = -2.90, *p* = 0.004, B–H critical value (c.v.) = 0.005. The analysis also indicated significant overall differences for depression change scores between the four cells that emerged from the interaction between type of play and role in the experiment (performed vs. simulated play X actor vs. spectator role), Kruskal–Wallis *H* (3) = 10.21, *p* = 0.017. Pairwise comparisons indicated a single difference that reached the statistical threshold, between the actors in the performed play and the actors in the simulated play, Mann–Whitney *U* = 8.00, *Z* = -3.06, *p* = 0.00222, B–H c.v. = 0.00227. Higher ranks were present for actors in the performed type of play (see Figure 2). No other difference emerged when comparing changes in emotional experience for all the other scales (*p*s > 0.05 or above B–H c.v.).

Next, we identified the most intense emotions that the participants experienced and the moments in the play that had generated them (using the three items we developed in this study).

First, we looked to see if the frequency of the most intense emotion reported by the participants during the artistic performance varied by the type of play or by the role assigned in the experiment (item 1). Non-parametric χ^2^-tests comparing the frequency of four categories of emotions (anger, fear, depression, and positive emotions), as reported across the types of play, the two roles, and the four cells resulted from their combination, showed no significant differences for any of these emotions (all *p*s > 0.05).

Item 2 asked participants to describe the moments in the play that had led to the most intense emotional experiences and the emotions that they have experienced. Emotions listed by participants were grouped under five categories: anxiety, depression, anger, disgust, and positive emotions. Non-parametric between-group comparisons and the B–H procedure applied to this outcome indicated no significant rank differences for the role in the experiment, type of play, and their combination (all *p*s > 0.05 or above B–H c.v.).

**FIGURE 3:**
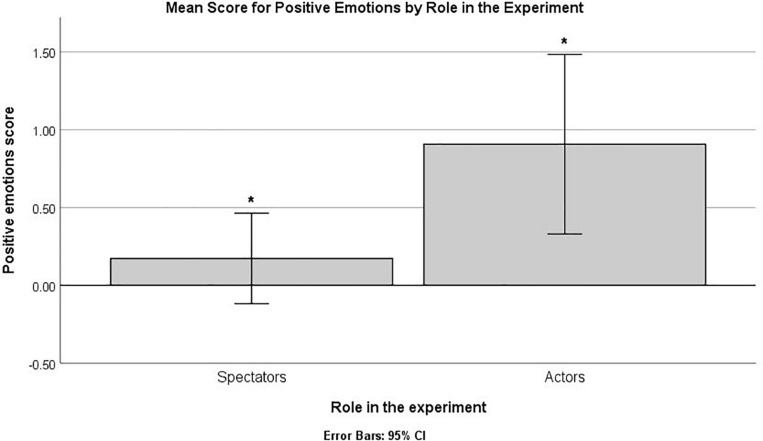
Mean frequency for positive emotions by role in the experiment (item 3). The bars marked by “*” indicate statistically significant differences based on a non-parametric analysis and B–H procedure for controlling false discovery rate (FDR).

When asked to report on the emotions generated by a prespecified list of moments selected from the plot (item 3), the actors more frequently reported that they experienced positive emotions, and this difference was statistically significant based on a Mann–Whitney non-parametric analysis, *U* = 122.00, *Z* = -2.41, *p* = 0.016, B–H c.v. = 0.025 (Figure 3). No differences were found for the type of play or the interaction effect (*p*s > 0.05 or above B–H c.v.).

### Effects of the Play on Presence

**FIGURE 4:**
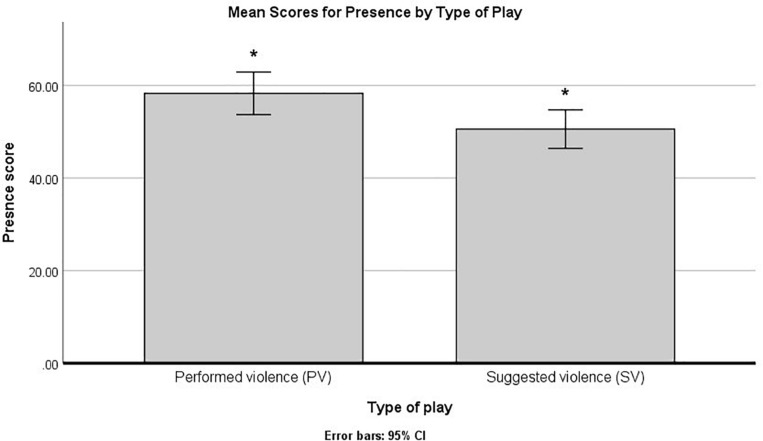
Mean scores for presence by type of play. The bars marked by “*” indicate statistically significant differences and B–H procedure for controlling false discovery rate (FDR).

In order to assess the presence that the participants experienced during the play, we conducted a two-way analysis of variance (ANOVA), using the type of play, role in the experiment, and their interaction as between-subject factors in the model. The results indicated a significant main effect for the type of play, *F*(1, 36) = 6.38, *p* = 0.016, η^2^*_p_* = 0.15, B–H c.v. = 0.025, but not for the role in the experiment, *F*(1, 36) = 3.56, *p* = 0.067, η^2^*_p_* = 0.09, B–H c.v. = 0.05, or for the interaction term, *F* (1, 36) = 0.82, *p* = 0.371, η^2^*_p_* = 0.02. The comparison of the estimated marginal means indicated that the significant effect was due to higher scores in the performed violence group, as compared to the simulated violence group (see Figure 4).

### Effects of the Play on Cognitive Processes

In order to assess the effects on cognitive processes, we analyzed the effects of the type of play, role in the experiment, and their combination, on performance in the word recall task. The dependent variables that we introduced in the analysis were the number of words under the violence category, the number of words opposed to this category, and the number of neutral words (not falling under any of the previous categories). We conducted a separate analysis for both true words recalled from the list, as well as for erroneous recalls (words falling under one of the above categories, but which were not on the list). This strategy yielded six dependent variables: real targets for violence-related words, real targets for words opposed to violence, real targets for neutral words, and fake recalls for each of them. The B–H procedure was applied across all these outcomes.

**FIGURE 5:**
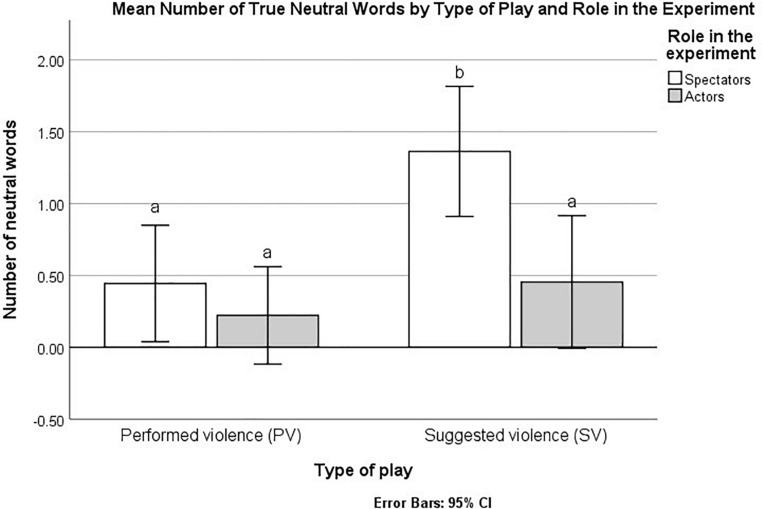
Mean frequency of true neutral words recalled by type of play and role in the experiment. Bars marked by different letters indicate statistically significant differences based on a non-parametric analysis and B–H procedure for controlling false discovery rate (FDR).

A significant overall difference emerged for neutral words when comparing all four cells representing the interaction between type of play and role in the experiment, Kruskal-Wallis *H* (3) = 14.08, *p* = 0.003. Comparing each pair of cells, we found (Mann–Whitney test) that spectators in the simulated play had a significantly higher ranks (correctly recalled more neutral words) than spectators in the performed violence play *U* = 16.50, *Z* = -2.69, *p* = 0.007, B–H c.v. = 0.005, actors in the simulated play, *U* = 22.50, *Z* = -2.65, *p* = 0.0080, B–H c.v. = 0.0083, and actors in the performed violence play, *U* = 10.50, *Z* = 3.16, *p* = 0.002, B–H c.v. = 0.002 (Figure 5). No other comparison for neutral words was significant (*p*s > 0.05 or above B–H c.v.). In addition, no effect of type of play, role in the experiment, or omnibus comparison between all cells reached statistical significance for any of the other dependent variables (all *p*s > 0.05 or above B–H c.v.).

### Association Between Emotional Experiences and Cognition

We examined whether there is an association between the emotional experience of the participants and cognitive variables. Namely, we were interested to see if their emotional state at the end of play was associated with their performance in the memory recall task. For this analysis, we used the posttest score on all the PDA + subscales and the one extracted from the PANAS, and all the six scores derived from the memory task. None of the correlations reached the statistical threshold (all *p*s > 0.05 or above B–H c.v.).

## Discussion

Our hypothesis was that the representation of aggressive actions will have a stronger impact on the subjects performing a realistic theatrical representation of a violent action PV than on those involved in SV, and it was partially confirmed by our results. Subjects in the PV group reported significantly larger increases of scores on the depression scale of PDA, compared to subjects in the SV group. Moreover, performers in the PV group had a significantly larger increase on the depression scale than performers in the SV condition. A surprising result was that actors reported more positive emotions toward violent actions of the script.

Exhibiting aggressive behaviors in a performed-violence scenario influenced the emotional experience of actors more than that of the spectators and differentially activated the cognitive structures related with violence and aggression, with spectators in the SV condition remembering significantly more neutral words than actors in the experiment, both in the PV and SV conditions. In addition, the memory recollection of neutral words was associated with the type of performance, with SV spectators remembering more neutral words than PV spectators. Based on this result, we can assert that watching the recollected experience of violence has a lower effect in activating the cognitive schema related to violence than performing the aggressive action, seeing it, or recollecting it.

These findings suggest that the embodiment of perceptions and sensations associated with the actual representation of aggression might have a major impact at emotional and cognitive levels. Differentiated emotional and cognitive outcomes of PV and SV correspond to different levels of perceived immersion, with more realistic representations having a stronger impact on the participants. This explanation is supported by the results indicating that participants in PV were more immersed in the performance than participants in SV.

In addition, our findings point to an often disregarded issue of the effects of performed actions on performers. While extensive studies are dedicated to the effects of watching violence, very few explore the effects of performing fictional violence. Some previous data states the importance of this direction of research. A study with a cohort of 800 actors found that they are more affected by depression than the mean population (Maxwell et al., 2015). Some qualitative accounts attribute at least part of this effect to fictional experiences of violence, with actors reporting posttraumatic stress disorder (PTSD)-like symptoms in connection to their work, particularly with the repeated embodiment of scenarios involving rape, physical violence, grief, and suicide (Robb et al., 2018).

The most striking result is that actors reported the presence of positive affect in relation to aggressive actions in the representation. We can consider this result as an experimental support of the two-pedal model of aggression (Elbert et al., 2017, 2018). Qualitative observations point out that reactions of disgust and amazement would accompany the first readings of the most violent actions during rehearsals. After several cycles of rehearsals, actors would end up finding them amusing. Laughter can be seen as a coping mechanism employed to change negative emotions into positive ones. Laughter is cited as very present at rehearsals of texts with high levels of violence such as “Blasted” by Sarah Kane (Kane, 1995). The actress performing Cate states in her recollection of the rehearsals (Ashfield, 2015) that despite rape, cannibalism, and sadistic actions, laughter was a common presence: “*At the read-through, some people at the Court were saying that it would be so dark and hard to do. But it was the opposite in rehearsals: we were all laughing all the time*” (Ashfield, 2015).

Reactive aggression is described as the response to a threat, which leads to a level of high arousal accompanied by negative affect valences. Appetitive aggression describes violent actions accompanied by positive affect (Fontaine, 2007; Weierstall and Elbert, 2011). In the two-pedal model of aggression, Elbert states that through the repetition of aggressive behaviors, reactive aggression can change under the influence of coping mechanisms in appetitive aggression. Interviews with former war combatants offer support for the effects of repetitive aggressive acts. Former fighters describe how their initial repulse toward killing changed, after a habituation phase, into an increasingly positive affect (Elbert et al., 2017, 2018). Reactive aggression is determined by high levels of emotional arousal with a negative valence, such as fear, hostility, anger, and rage. Negative emotions driven by the aggressive act can be replaced by feelings of satisfaction, enjoyment, and thrill. Elbert proposes a biological substrate for this change, a possible release of endorphins, to compensate for negative states driven by a high arousal situation (Elbert et al., 2017, 2018).

The actors in the PV condition had significantly higher depression scores at the end of the representation, but they also experienced significantly more positive emotions toward the violent actions in the script. Positive emotions toward aggressive behavior might be attributed to a combined effect of the role in the experiment and to the repetition of the violent actions. While further experiments are needed to elucidate if these changes could have long-term effects on performer behavior, our experiment shows that some of the subjects report positive emotions toward performed aggressive actions. Further control would be needed to assess a direct link between repeating the actions and the occurrence of positive emotions.

The fact that the PV condition and being an actor had an overall higher impact on our participants across all outcomes (as compared with SV and being a spectator) is consistent with the idea that the observed effects are related with the embodiment of aggressive behavior. The more participants embodied aggressive behaviors (e.g., performing the action instead of just talking about it or seeing it), the stronger the consequences they experienced.

Our study is not without limitations. First of all, the small sample size meant that our statistical power was generally low, and thus, many relevant effects might not have been identified. Second, we mainly relied on self-report measures, which did not allow for exploring other relevant effects of the representation on the experience of the participants (e.g., psychophysiological reactions). Third, our script was complex and included several violent actions, together with other elements that might have altered the emotional responses of the participants (e.g., being bullied, not just being aggressive), and it is hard to point at the exact components responsible for the effects we observed. Moreover, despite our efforts to make the two types of play as equivalent as possible, the fact that the actors in one condition interacted physically and those in the other one just through eye contact might have altered the correspondence between the two conditions. Future studies should try to overcome these limitations by using larger samples and other types of outcomes, as well as try to develop representations that are less likely to be affected by possible confounding variables.

## Conclusion

Given its constant presence across human civilization, violence has taken its place in all artistic and cultural forms of expressions. As such, theater has a long history of representing violence, from antiquity to present times, and while it is supposed that the cathartic function of representing violence through tragedies would have a beneficial effect on spectators, there is little empirical evidence on the positive or negative impact of artistic representations of violence for the audience (Przybylski and Weinstein, 2019). While in most countries there are public restrictions consisting in ratings of violence in movies or depiction of violent acts on TV, we are still very far from understanding the impact that violence representations have on the human brain and how we should deal with it at societal and personal levels. Strong data points to effects of media violence, including video games (Bushman and Anderson, 2001; Anderson et al., 2010) in determining increased aggressive behavior, but theatrical representations of violence could have therapeutic effects at individual and societal level. Theater practice is a very important form of complex human communication, based on cooperation. While dramatic theater has action at its core, and consequently representation of conflict, in the second half of the last century, new forms of postdramatic structures turned away from conflict (Heim, 2016). Shifting form conflict representation to postdramatic structures enhances the social functions of theatrical representation, like empathy development, community building, experience sharing, and collective thinking.

Regarding the theatrical esthetic traditions of representing or not representing violence, corresponding to the Greek and Roman theater, respectively, our data suggest that each of them has differentiated effects at emotional and cognitive levels, for both spectators and performers. Recollecting violence on stage might have a minimal effect, suggesting the possibility of distancing by both performers and spectators. The explicit depiction of violent actions in a realistic manner has a stronger emotional load. Moreover, performing violent acts might even increase positive emotions toward aggression, as our actors experienced positive emotions toward such acts. This result could be explained by the two-pedal model of aggressive behavior developed by Elbert et al. (2017, 2018). While our experiment does not provide information on the long-term effects of representing violent actions, further studies are required to analyze this phenomenon.

The practice of theater therapies seems to acknowledge that performing aggressive behavior is a dangerous game. Most interventions aimed at reducing aggressive behavior work with symbolic actions and imaginary objects – as in Bergman’s drama therapy method (Bergman and Hewish, 2015). Using dramatic metaphor in the therapeutic process lies at the core of drama therapy practice (Emunah, 2009; Landy, 2009). Brecht (1949) introduced the concept of distance in his political theater. The concept was further developed by Landy in his roleplay drama therapy system (Landy et al., 2003; Landy, 2009). The idea of distancing from fictional action is present in philosophers’ thoughts on theater, from Plato to Rancière (2008), and this points to the need for an active form of spectatorship, while showing philosophers’, theater makers’, and therapists’ constant preoccupation for separating action from its emotional load.

Anthropological studies on many societies have documented highly sophisticated performative scenarios for regulating aggressive behavior, sometimes performed in highly ritualized theatrical setups, like the courts of Kogu, song duels of the indigene populations of Greenland and Alaska, and all sorts of battles, which forbid killing or harming (Schechner, 2003). While generating new social rituals is beyond a realistic scope, by using the intrinsic power of theatrical play, grounded in scientific research, we could have a strong impact at the individual and perhaps societal level, by providing tools for promoting the self-control of aggressive behaviors. Theater has a long history of therapeutic interventions in modern times, starting with Moreno’s psychodrama. The idea of using theater for promoting adaptive behaviors, especially for regulating violence and aggression, spans a history of more than 30 years, with examples such as the Geese Theater Company that works with offenders. However, strong empirical evidence is still needed in order to prove the beneficial effect of using theatrical representations for this purpose (Bergman, 2009). Our study is a starting point for an empirical and experimental research program that would investigate the use of theater to understand and manage violent behavior.

## Data Availability Statement

The datasets generated for this study are available on request to the corresponding author.

## Ethics Statement

The studies involving human participants were reviewed and approved by the Ethics Committee of Clinical Psychology and Psychotherapy, Babeş Bolyai University, Cluj-Napoca, Romania. The patients/participants provided their written informed consent to participate in this study. Written informed consent was obtained from the individuals for the publication of any potentially identifiable images or data included in this article.

## Author Contributions

AB, BM, and SM participated in designing the experiment and conducting the experiment. AB wrote the text for the experiment and conducted the rehearsal process. SM analyzed the data. All authors participated in interpreting results and writing process.

## Conflict of Interest

The authors declare that the research was conducted in the absence of any commercial or financial relationships that could be construed as a potential conflict of interest.

## References

[B1] Anderson C. A. Shibuya A. Ihori N. Swing E. L. Bushman B. J. Sakamoto A. (2010). Violent video game effects on aggression, empathy, and prosocial behavior in Eastern and Western Countries: a meta-analytic review. *Psychol. Bull.* 136 151–173. 10.1037/a0018251 20192553

[B2] Aristotel (1957). *Poetica Translated by Balmuş C. Scientific Edition, Bucharest*. Available online at: http://epistematic.blogspot.ro/2013/03/aristotel-poetica.html

[B3] Ashfield K. (2015). *Kate Ashfield on Sarah Kane’s Blasted: The Whole Run was Charged With Energy*. Available online at: https://www.theguardian.com/stage/2015/jan/12/kate-ashfield-sarah-kane-blasted

[B4] Baim C. (2007). Are you a cognitive psychodramatist? *Br. J. Psychodrama Sociodrama* 22 23–31.

[B5] Baim C. Burmeister J. Maciel M. (2007). *Psychodrama: Advances in Theory and Practice.* London: Routledge.

[B6] Bandura A. (1983). “Psychological mechanism of aggression,” in *Aggression: Theoretical and Empirical Reviews*, Vol. 1 eds Geen R. G. Donnerstein E. I. (New York: Academic Press), 1–40.

[B7] Bandura A. (2001). Social cognitive theory: an agentic perspective. *Annu. Rev. Psychol.* 52 1–26. 10.1146/annurev.psych.52.1.1 11148297

[B8] Barsalou L. W. (2008). Grounded cognition. *Annu. Rev. Psychol.* 59 617–645. 17705682 10.1146/annurev.psych.59.103006.093639

[B9] Benjamini Y. Hochberg Y. (1995). Controlling the false discovery rate: a practical and powerful approach to multiple testing. *J. R. Stat. Soc. Ser. B (Methodological)* 57 289–300. 10.1111/j.2517-6161.1995.tb02031.x

[B10] Bergman J. (2009). “The bergman drama therapy approach: creating therapeutic communities in prisons,” in *Current Approaches In Drama Therapy*, 2nd Edn, eds Johnson D. R. Emunah R. (Springfield, IL: Charles C. Thomas Publisher), 330–354.

[B11] Bergman J. Hewish S. (2015). *Challenging Experience An Experiential Approach to the Treatment of Serious Offender.* Oklahoma City, OK: Wood N Barnes Publishing & Distribution.

[B12] Berkowitz L. (1990). On the formation and regulation of anger and aggression. A cognitive neo-associations analysis. *Am. Psychol.* 45 494–503. 10.1037/0003-066x.45.4.494 2186678

[B13] Berkowitz L. (1993). Pain and aggression: some findings and implications. Special issue: the pain system: a multilevel model for the study of motivation and emotion. *Motiv. Emot.* 17 277–293. 10.1007/bf00992223

[B14] Brecht B. (1949). *Kleines Organon für das Theater Sinn und Form Berlin*. Available online at: https://www.oxfordreference.com/view/10.1093/oi/authority.20110 803100039729

[B15] Brown S. Cockett P. Yuan Y. (2019). The neuroscience of Romeo and Juliet: an fMRI study of acting. *R. Soc. Open Sci.* 6:181908. 10.1098/rsos.181908 31032043 PMC6458376

[B16] Bushman B. J. Anderson C. A. (2001). Media violence and the American public: scientific facts versus media misinformation. *Am. Psychol.* 56 477–489. 10.1037//0003-066x.56.6-7.477 11413871

[B17] DiGiuseppe R. Tafrate R. C. (2007). *Understanding Anger Disorders.* Oxford: Oxford University Press.

[B18] DiLorenzo T. A. Bovbjerg D. H. Montgomery G. H. Valdimarsdottir H. Jacobsen P. (1999). The application of a shortened version of the profile of mood states in a sample of breast cancer chemotherapy patients. *Br. J. Health Psychol.* 4 315–325. 10.1348/135910799168669 30467716

[B19] Dollard J. Miller N. E. Doob L. W. Mowrer O. H. Sears R. R. (1939). *Frustration and Aggression.* New Haven, CT: Yale University Press.

[B20] Elbert T. Moran J. Schauer M. (2017). Lust for violence: appetitive aggression as a fundamental part of human nature. *e-Neuroforum* 23 77–84. 10.1515/nf-2016-A056

[B21] Elbert T. Moran J. Schauer M. (2018). Two pedals drive the bi-cycle of violence: reactive and appetitive aggression. *Curr. Opin. Psychol.* 19 135–138. 10.1016/j.copsyc.2017.03.016 29279212

[B22] Ellis A. (1994). *Reason and Emotion in Psychotherapy* (Rev. ed.). Secaucus, NJ: Birch Lane.

[B23] Emunah R. (2009). “The integrative five phase model of drama therapy in current approaches,” in *Drama Therapy*, 2nd Edn., eds Johnson D. R. Emunah R. (Springfield, IL: Charles C. Thomas Publisher), 37–64.

[B24] Eysenck M. W. Byrne A. (1994). Implicit memory bias, explicit memory bias, and anxiety. *Cognit. Emot.* 8 415–431. 10.1080/02699939408408950

[B25] Fontaine R. G. (2007). Disentangling the psychology and law of instrumental and reactive subtypes of aggression. *Psychol. Public Policy Law* 13 143–165. 10.1037/1076-8971.13.2.143

[B26] Girard R. (1995). *Violence and the Sacred.* Bucharest: Nemira.

[B27] Grotowski J. (2002). *Towards a Poor Theatre.* New York, NY: Routledge.

[B28] Heim W. (2016). Theatre, conflict and nature. *Green Lett.* 20:290303 10.1080/14688417.2016.1192000

[B100] Heinrichs A. (2000). Drama and dromena: bloodshed, violence and, sacrificial metaphor in euripides. *Harv. Stud. Class. Philol.* 100, 173–188. 10.2307/3185214

[B29] Huesmann L. R. (1998). “The role of social information processing and cognitive schema in the acquisition and maintenance of habitual aggressive behavior,” in *Human Aggression: Theories, Research, and Implications for Social Policy*, eds Geen G. R. Donnerstein E. D. (New York: Academic Press), 73–109. 10.1016/b978-012278805-5/50005-5

[B30] Janis I. King B. (1954). The influence of role playing on opinion change. *J. Abnorm. Soc. Psychol.* 49 211–218. 10.1037/h0056957 13151774

[B31] Kane S. (1995). *Blasted Royalcourttheatre.com.* London: Royal Court Theatre Productions Limited.

[B32] King B. T. Janis I. L. (1956). Comparison of the effectiveness of improvised versus non-improvised role-playing in producing opinion changes. *Hum. Relat.* 9 177–186. 10.1177/001872675600900202

[B33] Landy R. (2009). “Role theory and the role method of drama therapy,” in *Current Approaches In Drama Therapy*, 2nd Edn, eds Johnson D. R. Emunah R. (Springfield, IL: Charles C. Thomas Publisher), 65–88.

[B34] Landy R. J. Luck B. Conner E. McMullian S. (2003). Role profiles: a drama therapy assessment instrument. *Arts Psychother.* 30 151–161. 10.1016/S0197-4556(03)00048-0

[B35] Maxwell I. Seton M. Szabó M. (2015). The australian actors’ wellbeing study: a preliminary report. *Perform. Arts Med. Med. Problems* 13 69–113. 10.21091/mppa.2020.2012 32479582

[B36] McNamara T. P. (2005). *Semantic Priming: Perspectives from Memory and Word Recognition.* New York: Taylor & Francis Group.

[B37] Moran J. K. Weierstall R. Elbert T. (2014). Differences in brain circuitry for appetitive and reactive aggression as revealed by realistic auditory scripts. *Front. Behav. Neurosci.* 8:425. 10.3389/fnbeh.2014.00425 25538590 PMC4260506

[B38] Morin O. Acerbi A. Sobchuk O. (2019). Why people die in novels: testing the ordeal simulation hypothesis. *Palgrave Commun.* 5 1–10. 10.1057/s41599-019-0267-0

[B39] Neely J. H. (1991). “Semantic priming effects in visual word recognition: a selective review of current findings and theories,” in *Basic Processes in Reading, Visual Word Recognition*, eds Besner D. Humphreys G. W. (Mahwah, NJ: Lawrence Erlbaum Associates), 264–336.

[B40] Nevitt L. (2013). *Theatre and Violence.* New York: Palgrave Macmillan.

[B41] Niedenthal P. M. Barsalou L. W. Winkielman P. Krauth-Gruber S. Ric F. (2005). Embodiment in attitudes, social perception, and emotion. *Pers. Soc. Psychol. Rev.* 9 184–211. 10.1207/s15327957pspr0903_1 16083360

[B42] Opris D. Macavei B. (2005). The distinction between functional and dysfunctional negative emotions: an empirical analysis. *J. Cognit. Behav. Psychother.* 5 181–195.

[B43] Opris D. Macavei B. (2007a). The profile of affective distress; norms for the romanian population. *J. Cognit. Behav. Psychother.* 7 139–158.

[B44] Opris D. Macavei B. (2007b). “The profile of affective distress,” in *Sistem de Evaluare Clinicã (Clinical Evaluation System)*, ed. David (Coord.) D. (Cluj-Napoca: RTS).

[B45] Peachin M. (ed.) (2011). *The Oxford Handbook of Social Relations in the Roman World.* Oxford: Oxford Press.

[B46] Przybylski A. K. Weinstein N. (2019). Violent video game engagement is not associated with adolescents’ aggressive behaviour: evidence from a registered report. *R. Soc. Open Sci.* 6:171474. 10.1098/rsos.171474 30891250 PMC6408382

[B47] Rancière J. (2008). *Le Spectateur Émancipé.* Paris: La Fabrique Editions.

[B48] Robb A. E. Due C. Venning A. (2018). Exploring psychological wellbeing in a sample of Australian Actors. *Aust. Psychol.* 53 77–86. 10.1111/ap.12221

[B49] Schechner R. (2003). *Performance Theory.* New York: Routledge.

[B50] Sommerstein A. H. (2010). *The Tangled Ways of Zeus and other studies in and around Greek Tragedy.* Oxford: Oxford University Press.

[B51] Vannucci M. Nocentini A. Mazzoni G. Menesini E. (2012). Recalling unpresented hostile words: false memories predictors of traditional and cyberbullying. *Eur. J. Dev. Psychol.* 9 182–194. 10.1080/17405629.2011.646459

[B52] Watson D. Clark L. A. (1999). *The PANAS-X: Manual for the Positive and Negative Affect Schedule-Expanded Form.* Iowa City, IA: University of Iowa.

[B53] Weierstall R. Elbert T. (2011). The appetitive aggression scale-development of an instrument for the assessment of human’s attraction to violence. *Eur. J. Psychotraumatol*. 2. 10.3402/ejpt.v2i0.8430 PMC3402137 22893817

[B54] White C. N. Kapucu A. Bruno D. Rotello C. M. Ratcliff R. (2014). Memory bias for negative emotional words in recognition memory is driven by effects of category membership. *Cogn. Emot.* 28 867–880. 10.1080/02699931.2013.858028 24303902 PMC4350772

[B55] Witmer B. G. Singer M. J. (1994). *Measuring Immersion in Virtual Environments.* (ARI Technical Report 1014). Alexandria, VA: Army Research Institute for the Behavioral and Social Sciences.

[B56] Witmer B. G. Singer M. J. (1998). Measuring presence in virtual environments: a presence questionnaire. *Presence* 7 225–240. 10.1162/105474698565686 32495221

