# Supplementary material for: Emotional and Cognitive Responses to Theatrical Representations of Aggressive Behavior
Source: Front Psychol. 2020 Aug 14;11:1785. doi: 10.3389/fpsyg.2020.01785 (PMC7456896; doi:10.3389/fpsyg.2020.01785)
Supplement: Supplementary file 3 [file Data_Sheet_1.pdf]

Emotional and cognitive responses to theatrical representations of aggressive behavior

***Alexandru I. Berceanu [1], Silviu Matu[2], Bianca Macavei[3]**
[1]Laboratory for Cognitive Development and Applied Psychology through Immersive Experiences, CINETic Centre, UNATC IL Caragiale, Bucharest, Romania

[2]Department of Clinical Psychology and Psychotherapy, Babeș-Bolyai University, Cluj-Napoca, Romania

[3]Department of Psychology, Babeș-Bolyai University, Cluj-Napoca, Romania

Correspondence:

berceanu.cinetic@unatc.ro

Supplementary Materials
Scripts of theatrical interactions

Colleagues

developed by Alexandru I. Berceanu

Script 1. Performed aggressive action (PV)

Situation 1. *Victor is at work, he has a food casserole and a fork, he opens the casserole. He also has a steaming tea. His phone rings.*

Victor: Hey! Oana, no, I didn eat it yet. Now. Just now I'm tasting it it. It's better than the last time. I didn't have the time yet, we have to finish the project. The paella is amazing–it's even better than the last time. Dan said we should give the material today, he is crazy. As always, I work and at the end he brags about it. Don't wait for me, I'll get home in the morning. My head hurts so bad already, I told you, it's like it's throbbing. Yeah, thank you. I'm lucky to have it, the screen is cool, it's super-fast, I was sick of that old computer. Yes. Wait, he is coming, this is prick… I'll call you back.

Situation 2. *Dan is coming back into the office.*

Dan: How are you, *Foodie*? Is the porridge good? Let me see how Oana made it this time. She fucks as well as she cooks?

He takes Victor's fork and takes a bite from Victor's food. He looks at what Victor is working.

Dan: You stupid, what is this? It's all wrong! What did I tell you: you should put at D4? 150.000! You're so stupid, your brain is the size of a rice grain. Redo the column, print the plan and redo all the folder. I hope you finish the job until the morning! It must come out perfect, no mistake is tolerable.

The work phone rings, Dan answers.

Dan: Yes, boss. Everything is under control. Yes, *Foodie* is also here, he is smiling at you. He has something to redo.

Victor gets up and goes out of the room.

Dan: No, it won't happen again, we will check three times and at the end one more time and we'll finish on time for the City Hall. No, boss, it's not that I like the money, but the bank loan is big! I'll motivate Victor as well. He is good, but he lacks motivation, he needs some pushing. He doesn't care about the money, but I know where I should kick him to make him dance. Yes, to you too.

He puts the phone down, looks at Victor's food, starts to eat while working on the computer. Victor comes in.

Victor: Someone broke the plotting machine.

Dan: What do you mean?

Victor: Someone forced the helix head.

Dan: Forced what?

Victor: The… thingy.

Dan: It should work, restart it, it should work! I said everything would be ready until tomorrow morning! And you need also redo the work! You made a mistake, you redo it!

Victor: You broke it, you always force it!

Dan: How dare you! Hey, *Foodie*, you should see how I will force your face if you don't start the plotting machine. Why the hell are you even here if you can't even start the plotting machine?

Victor sees that there is no more food in the food casserole. Victor lets the plans to fall on the floor.

Victor: I can't work like that, I must get some rest, my blood sugar drops, I can't think like that, I go home. I can't do it anymore; I don't care what the boss will say.

Victor slowly sits on a chair.

Dan: You wanker! Warm! You think that because you are weak, we will lose this project? I will make you work! You can't really get upset because of some food, what are you, a pussy? You need balls for this! You're a wanker and I will make you work! Impotent wanker! I'll hit you until you get up. Victor, Vicky! Hey, turn that shit up, I will get you something from Mc to get you blood sugar up.

Victor: I don't eat that shit!

Dan: Vic get up! Vic! That's not the way the champs do it, the champions resist! Come on, get up, time to exercise!

Dan throws a small ball towards Victor, Victor lets it slip.

Dan: Vic, Vicky wake up, we can't sleep.

Dan slaps him like a trainer would, but pretty hard.

Victor: Stop it!

Dan: Come on, hit me, be a man! Come on, come on, Foodie.

Dan hits him several times, provokes him, Victor can't refrain and hits Dan once. Dan jumps on him, Victor grabs an object to defend himself.

Dan: That's good, Foodie, you are up! Come on, get to work, I'll go get something from McDonald's for you. If you can't make the plotting machine work, I will call our boss and tell him to send here a plotter from somewhere else or send someone to repair it, I don't think he went to sleep yet. We must get this project done, so, in order to avoid any crazy ideas, you might have, you should give me your key. Give it to me!

Dan starts looking through his things; he takes the key from Victor's bag.

Victor: No need to do this, don't, Dan, don't be stupid.

Dan: We really can't afford to run to sweet Oana.

Dan gets out, closes Victor in with a key. Victor wants to tear the plans, puts his hands on his head and stops. He takes the phone out.

Victor: Hi, no I am not done yet, that idiot ate my food. He is an idiot, he is not able to do anything, he even broke the plotting machine. I don't know what I'm going to do to him. I can't stand him. No, nothing happened. No, I'm not coming. We must finish the project. I feel like crying of frustration. No, I'm not going to hit him in the head, though I feel like it. I will mess up this project or I will send an email with porn from his email address to our HR colleagues. I will mess up his project. His ass is so desperate for money, this is what will hurt him! Sure. I can't talk, I must go. Yes, I will take care. You know me when I am tired, I feel like I'm going crazy. Especially when I know that I do everything, and he takes credit for it. I do everything, he just destroys it. I can't stand the humiliation; I'm nobody's slave. I feel sick when I see him, this can't go on like that.

Victor goes to Dan's computer, works something there.

Victor: I feel like smashing his head. He calls me Foodie! This should hurt him; this should burn him.

Dan comes in, Victor gets up from Dan's computer who gives him the food.

Dan: There you go, food for your belly! Who's your daddy? Did you manage to repair the plotter? It doesn't matter, boss will send someone in to fix it. There, I saved your ass one more time. Who's your daddy? What's this? Have you gone mad? Why did you stick your nose in here? Is this a joke? What, you completely erased it? You have no right to touch my computer. How dare you? Give me the computer.

Victor is laughing. Dan takes Victor's laptop, works on it, looks for the project in Victor's computer.

Dan: You're an imbecile.

He takes the laptop and repeatedly hits it on the desk.

Dan: Redo everything, start now to redo it. You have to finish everything in four hours. Plotting included. With plotting, get it? You don't leave, this is where you die. You're an animal. This is not how one works. You're an animal. We must deliver the project on time.

Victor: Stop, it's new. It's from Oana.

Victor was sharpening a pencil and he sticks it in Dan's arm. Dan screams in pain. Victor throws the hamburger and coffee on Dan, the coffee is hot.

Victor: Enjoy!

Victor turns the light out and gets out. Dan is heard moaning in the dark.

Victor: Daddy!

The colleagues 2. Suggested aggressive action (SV)

Situation 1.

Victoria: It was the third day of overtime working, Dana, the imbecile, constantly says we can do this, she only cares that her salary goes up. She has a bank loan and she needs more money. She always brags with my work as if she did it. I work, she brags. My head spins because I didn't get enough sleep, I can't stand to work tired. I wanted to go home, Sergiu had given me a gift, a brand-new laptop and I wanted to see how cool it was. It was perfect timing; he knew that I couldn't stand the old junk. I had just taken the food from the microwave, it was 9 in the evening, and I didn't even have the time to get a bite all day long. I had finally relaxed a little bit and gotten used to the idea that horror does nothing but takes all the credit.

Situation 2.

Dana: I came into the office, the sucker was eating while I had been running all day long the entire city for different papers, I got the last documents at 10 minutes to 9 in the evening, and she was relaxing in front of the computer with hot food in front. She is not capable of doing anything without me. It could hear my stomach growling, so I pretended I was joking and took a large spoon from her food. If you want to keep someone under pressure you can't say you haven't eaten all day long, since this would diminish the respect. I take a look at the text and on the 4th column: big mistake. I knew for sure that we had established what we put there. I understand that you must eat, but after that you verify that the work you are doing is impeccable. But the idiot doesn't care about mistakes; she doesn't care about promotions or money. She doesn't have a bank loan and her boyfriend gives her, as a gift, a laptop, just like that. It wasn't even her birthday. I make her do the work again, I call our boss, I cover the idiot's ass by not saying a word. She is always on the verge of quitting and I have to

keep the team together, it's not really the most pleasant thing, but even after I save her ass, she looks at me like I'm a terrorist. If she is weak, then she must be hustled.

Victoria: I enter the office and I can her bragging on the phone, more than that, humiliating me. She called me a worm. Incapable as a worm. If she never does anything, she, of course, is never wrong. She only talks and talks. And I'm sure she is the one who broke the plotting machine, it's broke. I think she could break a door with a remote control that's how bad she is with objects. The button on the plotting machine was broke, if you push the button as a desperate person it gets stuck. Dana is desperate like that, Dana broke the button. And she ate my food. A person can't take that much. If you are hungry you buy food or, if it's the case, ask for food, where would we get if everybody takes what's not theirs? We'd be stealing everyone blind.

Action: We only see how Dana eats Victoria's food, Victoria moans with each bite.

Situation 3.

Victoria: When my blood sugar goes down, I cannot think anymore, first I feel very nervous. I feel like hitting something. When I saw the empty food casserole, I felt like screaming. I broke down. The plans fell on the floor and I felt a weakness in my knees. I felt like I couldn't even breathe so I lay down. The jerk told me she would get food from McDonald's, Mc Crap. Why didn't she took something for herself from Mc? I wasn't able to do anything; I only wanted to go home.

Dana: She started acting like a brat, she was almost fainting, what a phony! So, she makes a mistake and then she faints. It does not work like this. I can't take the abandonment and weak and lazy people and, moreover, we made a commitment that we would finish the project. I had to activate her, to get her all riled up. If I do so, then she works excellent, the greatest projects come from working under stress. I threw her the ball, she didn't even react, so I took it no the next level, slap on her face

She shows how.

Just like at box training, you must arouse her senses. She reacted immediately. At the beginning she avoided it, but with a little pressure and two more slaps she woke up. To hell with her, I could not imagine she has such a powerful slap, like a shepherd. If she never slapped someone in her life she has no idea how to do it. She hit me with her bones, it really hurt, my face was burning.

Action: Dana hits the desk with her hand. The two are at a distance one from the other. Victoria's head tilts to one side, then to the other, this is repeated several times, Victoria hits the desk once, but hard. Dana screams.

Situation 4.

Victoria: When I felt the first slap, my lungs stopped, I felt like something was blocking my throat, I was ready to burst in tears. My hand just flew towards her face. It must have hurt her, I felt it hurt her because my hand also hurt. I saw in her eyes that she was scared of me. I felt released, she instantly put the tail between her legs. She left to get me some food, or that's what she said, she felt because she was afraid.

Dana: She woke up. It was clear she woke up; my face was hurting. Badly. I didn't believe that that sucker has that much force. I went to get her some food and I left her

so she would come to her sense, but I took her key. She did protest a little bit, but that's all.

Victoria: I talked to Sergiu on the phone, I didn't tell him that she locked me in, it was to humiliating. The thought that the door was locked made me sick, I felt like a scum. I could have left, force the doors, but that seemed embarrassing. I was humiliated. I had to do something to hurt her. More than a slap. The project.

Situation 5.

Dana: I had to lock her in. She is so weak; she would be ready to run home in the second I left. I bought food for her, on the way back I called my boss and I told him to send someone to repair the plotting machine, I told him it's wasn't working properly anyway. Again, I covered her, and I didn't tell him she made a mistake. When I came back, no thank you, no nothing! Of course, she did not repair the plotter. I opened the project to verify it once again and nothing, nothing in the computer, I thought that she made a joke maybe she changed the name of the file, but when I looked at her and I knew she erased it. An idiot, that's not how you work, she has laughing like a stupid cow. I think that's exactly when she erased it from her computer as well. She made it on purpose so to destroy me. There is no reason to do something like this, no explanation. I tried to look for it in her new shitty computer and I saw her laughing and I took the computer and hit it on the table. And again, and again. I felt my anger was fading away.

The action is dilated, we see Dana throwing Victoria's food on the computer then she takes her laptop and repeatedly hits it on the desk.

Dana: Obviously her smile disappeared. I think I could see tears in her eyes. I told her she won't leave until she makes it again. It was only normal. If she called the Police, I think even they would get me.

Victoria: I don't know what happened, I cannot understand it. In fact, I know, I had the pencil in my hand, I had just sharpened it, I couldn't take it, I stabbed her in the arm with my pencil. I felt it go in, she screamed like an animal. She had the guts to ask me why I did that; I said I didn't like McDonald's. I threw the hamburger in her head and then the coffee, I didn't think it was hot, she screamed like an animal. I am nobody's slave.

The action is dilated: Victoria stacks the pencil in the air. Dana puts her hand on her arm, there is blood flowing, she is silently screaming. Then she throws the coffee on Dana, we see coffee dripping from Dana.
